# A Two‐Stage Plasma Biomarker Algorithm Reduces Amyloid PET Use and the p‐Tau217 Gray Zone

**DOI:** 10.1002/acn3.70560

**Published:** 2026-10-19

**Authors:** Harthik Parankusham, Casey Vanderlip, Colin Birkenbihl, Eashwar Krishna, Chizobam Ugboaja, Andrew E. Budson, Brandon Frank, for the Alzheimer's Disease Neuroimaging Initiative

**Affiliations:** ^1^ Department of Physiology and Neurobiology, UConn Computational Biology Core University of Connecticut Storrs Connecticut USA; ^2^ Department of Neurobiology and Behavior University of California Irvine Irvine California USA; ^3^ Department of Neurology, Massachusetts General Hospital Harvard Medical School Boston Massachusetts USA; ^4^ Department of Molecular and Cell Biology University of Connecticut Storrs Connecticut USA; ^5^ Yale School of Medicine, Yale New Haven Health New Haven Connecticut USA; ^6^ VA Boston Healthcare System Boston Massachusetts USA; ^7^ Alzheimer's Disease Research Center Boston University Chobanian and Avedisian School of Medicine Boston Massachusetts USA

**Keywords:** Alzheimer's disease, amyloid PET, cognitively unimpaired, gray zone, p‐Tau217

## Abstract

**Objective:**

Plasma p‐Tau217 leaves 30%–50% of patients in an indeterminate “gray zone” often requiring Aβ‐PET. We developed GRAD (Gatekeeper‐Reflex for Alzheimer's Disease), a two‐stage Aβ‐positivity prediction model to improve classification while reserving Aβ‐PET for residual uncertainty.

**Methods:**

GRAD was developed in 145 cognitively impaired ADNI participants and externally validated in 1644 cognitively unimpaired A4/LEARN participants. Stage 1 used p‐Tau217 alone; probabilities between 0.25 and 0.75 were considered indeterminate. Stage 2 used L2‐regularized logistic regression combining plasma‐biomarkers, genetic and demographic features. Aβ‐PET served as the reference standard (ADNI florbetapir SUVR > 1.11; A4 Centiloid ≥ 20).

**Results:**

Full model AUC was 0.915 (95% CI, 0.859–0.961) internally and 0.879 (95% CI, 0.861–0.895) externally. In the external gray zone (*n* = 630), Stage 2 improved discrimination over p‐Tau217 alone (AUC 0.793 vs. 0.719; *p* < 0.001), increased correct classification without PET from 41.4% to 56.2%, and reduced PET referral from 35.9% to 24.3%. In the p‐Tau181 subset (*n* = 256), Stage 2 outperformed p‐Tau181 (AUC 0.799 vs. 0.597; *p* < 0.001). Overall, 89.0% of ADNI and 90.7% of A4/LEARN participants were classified without PET; modeled testing costs were 44%–52% lower than p‐Tau217 screening with PET confirmation of the gray zone.

**Interpretation:**

Reflex multimarker testing improves indeterminate p‐Tau217 classification while retaining Aβ‐PET for residual uncertainty. GRAD may reduce PET use and testing costs in memory‐clinic triage, prevention‐trial enrichment, and disease‐modifying therapy workup.

## 1 Introduction

Blood‐based biomarkers (BBMs) for Alzheimer's disease (AD) enable detection of cerebral amyloid‐β (Aβ) pathology without the cost and limited accessibility of Aβ‐positron emission tomography (Aβ‐PET) or the invasiveness of cerebrospinal fluid (CSF) analysis [1, 2, 3].

Recently, phosphorylated tau217 (p‐Tau217) has exhibited the highest accuracy in plasma for identifying Aβ‐positive (Aβ+) individuals [4, 5, 6, 7, 8, 9, 10], even in the earliest stages of disease [11, 12, 13]. In clinical practice, p‐Tau217 plays the crucial role of screening individual eligibility for prevention studies and recent FDA‐approved disease‐modifying therapies (DMTs) [3, 14].

However, for preclinical‐AD and DMT‐eligible individuals where treatment decisions are critical, there exists a large overlap of p‐Tau217 values in classifying Aβ + versus Aβ‐ [14, 15]. As a result ~30%–50% of individuals fall within a “gray zone” where p‐Tau217 alone cannot confidently classify Aβ‐status [5, 14, 15, 16]. Several studies have attempted to resolve this uncertainty, including staged p‐Tau217/Aβ‐PET workflows and complementary BBMs such as p‐Tau181 [16, 17, 18, 19, 20, 21]. However, these studies had modest sample sizes, lacked external validation, and did not specifically target MCI and preclinical AD—the populations most likely to benefit from prevention treatments. Although multimarker approaches incorporating p‐Tau217, Aβ42/40 [22, 23, 24, 25], and glial fibrillary acidic protein (GFAP) show promise [26, 27, 28], they have not yet been adequately tested within this gray zone population.

In this study, we developed the “Gatekeeper‐Reflex model for AD” (GRAD), a two‐stage plasma‐based algorithm to classify individuals in the gray zone for Aβ+, with the goal of reducing residual confirmatory Aβ‐PET scans. We aimed to: (i) assess GRAD internal and external performance in DMT‐eligible and pre‐clinical populations (ii) benchmark GRAD against existing methods to ameliorate the gray zone and (iii) simulate our model's health‐economic impact.

We hypothesized that a plasma‐based staged model—like reflex testing in laboratory medicine [29, 30]—could resolve gray zone uncertainty in DMT‐eligible and preclinical‐AD individuals. As such, GRAD is intended as a triage framework and not a replacement for either p‐Tau217 or confirmatory Aβ testing. The readout is an estimated probability of Aβ positivity that determines the next step in a staged diagnostic pathway: high‐confidence results are resolved using p‐Tau217 alone, indeterminate results undergo reflex testing with additional plasma, genetic, and demographic information, and cases that remain uncertain are referred for confirmatory Aβ‐PET. This framework could be applied wherever Aβ status informs downstream decisions, including memory‐clinic evaluation and disease‐modifying therapy workup, enrichment of prevention trials, and other settings in which universal confirmatory imaging is impractical.

## 2 Methods

### 2.1 Study Populations

We drew participants from two independent cohorts for model development and external validation. GRAD was developed in cognitively impaired participants and externally validated in cognitively unimpaired participants, thus allowing us to assess transportability across clinically distinct populations.

#### 2.1.1 Development and Training Cohort

We selected cognitively impaired participants from the Alzheimer's Disease Neuroimaging Initiative (ADNI) (*31*) whose plasma p‐Tau217 and Aβ‐PET were collected at the same time. The 145 individuals who met these criteria formed the development cohort: MCI (*n* = 117, 80.7%) and AD dementia (*n* = 28, 19.3%). We considered ADNI florbetapir SUVR > 1.11 [31, 32, 33] as Aβ + .

#### 2.1.2 Independent Validation Cohort

For the external validation cohort, we selected 1644 participants with baseline p‐Tau217 and Aβ status available from the Anti‐Amyloid Treatment in Asymptomatic Alzheimer's disease (A4) Study (1119 in A4, 525 in LEARN) [34, 35, 36, 37]. This included 1145 Aβ + and 499 Aβ‐ participants. We considered Centiloid ≥ 20 as Aβ + per the Centiloid standardization framework [35, 38].

### 2.2 Plasma Assays and Biomarker Acquisition

ADNI plasma p‐Tau217 was measured on two platforms: the Lumipulse G p‐Tau217 assay (Fujirebio) at the University of Pennsylvania (UPENN) in 81 of the 145 ADNI participants, and the Janssen p‐Tau217 assay in the remaining 64 [9]. Plasma samples were collected and stored as per ADNI biofluid protocols [39]. The UPENN panel provided GFAP in 81 participants, and Aβ42/40 in 80. The Janssen subset contributed p‐Tau217 only. The APOE genotype was available in 132 ADNI participants. APOE genotype was obtained from the ADNI APOE results file, and demographic data, diagnostic classification and florbetapir amyloid PET (AV45 SUVR) from ADNIMERGE. We also report analyte availability by cohort and platform (Table S1). All files were downloaded from the ADNI database (http://adni.loni.usc.edu/) under an approved data use agreement.

In A4, all 1644 participants had p‐Tau217 measured via the Lilly Research Laboratories MSD immunoassay [40]. GFAP and Aβ42/40 were measured on the Roche Elecsys platform in a subset of the cohort: GFAP in 931 participants, and Aβ42/40 in 919, with APOE genotype available in 1642. Aβ42/40 was available to 474 A4‐arm participants and 445 LEARN participants.

Participants missing an analyte were retained with the missing value imputed from the training‐set median (Section 2.3), so the denominators reported throughout include them. We ensured test‐set values were never imputed from the test cohort's own distribution. To assess the potential effect of missing analyte measurements and imputation on model performance, we additionally performed a complete‐panel sensitivity analysis restricted to participants with all model biomarkers available, with no imputation (Table S2).

### 2.3 Data Preprocessing and Harmonization

For preprocessing and harmonization, we Z‐normalized all biomarkers measured from blood plasma to a prespecified reference population of CU, Aβ‐negative individuals within each cohort and assay platform [8, 9] (Table S3). The raw biomarker values were log‐transformed and Z‐scores were computed as: *Z* = (log(1 + *x*) − *μ*_ref)/*σ*_ref. This preprocessing step was crucial since both cohorts utilized different assays for p‐Tau217 (Table S1).

The Aβ42/40 ratio was the only exception and entered our model as a raw log ratio. Since it is a ratio of two analytes measured on the same platform from the same sample, the platform scale factors cancel within it. Anchoring it to the reference could add noise without removing bias.

### 2.4 Design of the Two‐Stage Algorithm

The model consisted of two stages. Stage 1 (Gatekeeper) was a univariate logistic regression model using only p‐Tau217 to predict Aβ + individuals. We set classification thresholds based on p‐Tau217 predictive confidence: Aβ‐negative where *p* < 0.25 and Aβ‐positive where *p* > 0.75. Cases falling within the intermediate gray zone (0.25 ≤ *p* ≤ 0.75) [16] were triaged to the next stage (Figure 2A). In Stage 2 (Reflex), for gray zone cases, we implemented an L2‐regularized logistic regression (*C* = 0.1 set a priori to promote strong shrinkage; balanced class weights) on features standardized within each training fold (StandardScaler, scikit‐learn). The feature set comprised p‐Tau217, GFAP, a tau‐Aβ divergence ratio (log[p‐Tau217]—log[Aβ42/40]), log Aβ42/40, a GFAP by p‐Tau217 interaction term, age, and APOE ε4 carrier status [41] (Figure 1). We manually selected BBM interaction terms based on biological priors, instead of utilizing ML model‐based discovery which could create cohort dependence.

**FIGURE 1:**
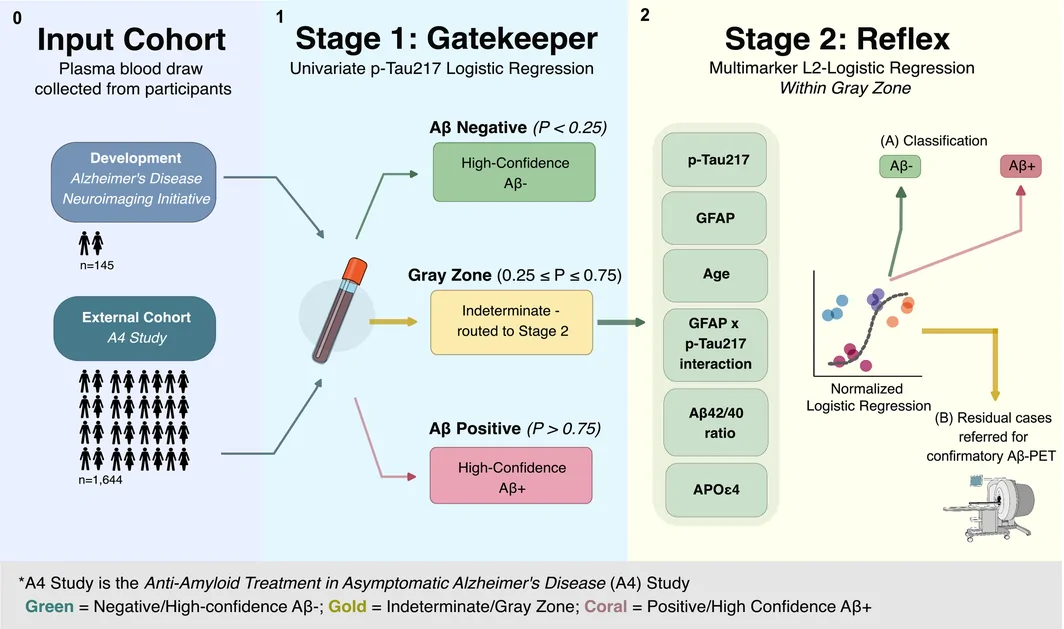
Study design and workflow. Participants from the development cohort (ADNI, *n* = 145 cognitively impaired) and the external validation cohort (A4 and LEARN, *n* = 1644 cognitively unimpaired) contributed a single plasma draw. In Stage 1 (Gatekeeper), a univariate logistic regression on harmonized p‐Tau217 returns a predicted probability of Aβ positivity: Participants below 0.25 are classified high‐confidence Aβ‐negative (green), those above 0.75 high‐confidence Aβ‐positive (coral), and those between 0.25 and 0.75 fall in the indeterminate gray zone (gold) and are triaged forward.

We considered, in both cohorts, reflex cases with predicted probability outside 0.40–0.60 resolved, while cases within 0.40–0.60 remained indeterminate and referred for confirmatory Aβ‐PET. These thresholds served different purposes: the wider Stage 1 band prioritized sensitivity for identifying cases warranting additional blood‐based testing, whereas the narrower Stage 2 band identified residual uncertainty warranting confirmatory Aβ‐PET.

### 2.5 Model Validation Strategy

All model development decisions, including feature specification, regularization strength, and classification thresholds, were established in the ADNI development cohort before external validation. Internal performance was estimated using leave‐one‐out cross‐validation (LOOCV), with data preprocessing, harmonization, standardization, and Stages 1 and 2 model fitting repeated within each training fold to prevent information leakage. We calculated percentile bootstrap confidence intervals (CIs; 2000 resamples of the LOOCV predictions) for AUC, sensitivity, and specificity. After internal evaluation and harmonization across assay platforms, the final ADNI‐trained model and prespecified decision thresholds were applied without refitting or optimization to the independent A4/LEARN cohort. A4/LEARN therefore served exclusively as an external validation dataset and did not inform model training, feature selection, or thresholding choices.

For A4 external validation (A4‐arm and LEARN), we performed a standard binary classification. We additionally derived a Spearman correlation between individual‐level predicted probabilities and Aβ‐PET centiloid values for a continuous validity check. We assessed calibration by the slope and intercept of observed outcome on the predicted log‐odds.

GRAD's statistical analysis consisted of AUC with bootstrap confidence intervals (2000 resamples), sensitivity, specificity, positive predictive value (PPV), negative predictive value (NPV), likelihood ratios (LR+, LR−) under STARD 2015 diagnostic guidelines [42], and Brier scores for calibration of probabilistic predictions. We only used DeLong's test [43] for paired comparisons of two models scored on the same participants. We compared classification accuracy between p‐Tau217 alone and GRAD Stage 2 on the same participants using McNemar's test.

### 2.6 Benchmarking Against p‐Tau217 and p‐Tau181

We benchmarked GRAD Stage 2, first against p‐Tau217 alone in the gray zone, then against p‐Tau181 integration—the single published BBM‐only workflow designed to address this problem [17]. Against p‐Tau217, in both cohorts, we compared gray zone AUC by DeLong's test and applied the 0.40–0.60 indeterminate band to both scores—Stage 2 versus p‐Tau217—to compare the proportions determined correctly or incorrectly and referred for PET. The paired reclassifications were assessed using McNemar's test. Against p‐Tau181, we assessed the same quantities in the 256 A4 gray zone participants with a p‐Tau181 measurement. Two considerations apply in our benchmarking analysis. First, benchmarking Stage 2 versus p‐Tau181 was limited to the preclinical A4 cohort, since only 17 of the 145 ADNI participants contained visit‐matched p‐Tau181 metrics. Second, we applied the same validation rule as the original p‐Tau181 integration (Giacomucci et al.) study: A dichotomous cutoff derived by Youden's index in the full cohort of impaired individuals. This meant no cases were referred to Aβ‐PET, while GRAD abstains from classifying significantly low‐confidence cases. Thus, we provide decision‐level metrics alongside AUC.

We conducted analyses in Python version 3.9.6 using scikit‐learn version 1.6.1, NumPy version 2.0.2, pandas version 2.3.1, SciPy version 1.13.1, and statsmodels version 0.14.6. All statistical tests were two‐sided and considered *p* < 0.05, statistically significant.

### 2.7 Cost Impact Simulation

To quantify GRAD's health economic impact, we developed a clinical decision‐based model comparing three diagnostic strategies for a simulated cohort of 10,000 patients: Universal Aβ‐PET (estimated conservatively $3000 per scan), p‐Tau217 screening + PET for gray zone cases ($350 per single‐analyte p‐Tau217 test + PET for all indeterminate cases), and our GRAD staged algorithm. GRAD is billed as a reflex assay: every patient receives the $350 p‐Tau217 test, the multi‐analyte add‐on panel ($250) is charged only to the fraction routed to Stage 2, and PET is reserved for the residual indeterminate cases. Resolution rates were derived from the observed LOOCV and A4 validation results (Sections 3.2, 3.3, and 3.5). We determined unit costs based on 2024 U.S. Medicare reimbursement schedules [44, 45, 46] and, where necessary, we assumed higher costs to avoid potential overestimation of savings (Table S4). We report cost per correct diagnosis, counting cases referred for PET as correctly diagnosed.

## 3 Results

### 3.1 Baseline Demographic Characteristics

The 145 participants from the ADNI cohort used for model development had a mean age of 73.3 ± 7.1 years, were 37.2% female, and 89.0% White (Table 1). 46.2% of participants with an available genotype were APOE ε4 carriers and the median p‐Tau217 level was 0.139 pg/mL, with 58.6% Aβ positive. A4/LEARN participants had a mean age of 71.5 ± 4.7 years, were 60.2% female; 47.4% were APOE ε4 carriers, the median p‐Tau217 level was 0.192 pg/mL, and 69.6% were Aβ positive (Table 1).

**TABLE 1:** Baseline cohort distribution and characteristics.

Characteristic	ADNI development cohort (*n* = 145)	A4 + LEARN validation cohort (*n* = 1644)
Age, years, mean (SD)	73.3 (7.1)	71.5 (4.7)
Female, *n* (%)	54 (37.2)	990 (60.2)
Education, years, mean (SD)	15.8 (2.7)	16.6 (2.7)
White, *n* (%)	129 (89.0)	1546 (94.0)
MMSE, mean (SD)	26.5 (3.3)	28.8 (1.2)
APOE ε4 carrier, *n* (%)^a^	61 (46.2)	779 (47.4)
Clinical status, *n* (%)		
Cognitively unimpaired	0	1644 (100)
Mild cognitive impairment	117 (80.7)	0
AD dementia	28 (19.3)	0
Aβ status		
Aβ‐PET positive, *n* (%)	85 (58.6)	1145 (69.6)
Positivity threshold	Florbetapir SUVR > 1.11	Centiloid ≥ 20
Plasma p‐Tau217		
Median (IQR), pg/mL	0.139 (0.067–0.356)	0.192 (0.142–0.276)
Assay platform	Lumipulse G, *n* = 81; Janssen, *n* = 64	Lilly Research Laboratories MSD
A4 randomized arms	—	1119 (68.1)
LEARN	—	525 (31.9)
Panel analyte availability, *n* (%)		
GFAP	81 (55.9)	931 (56.6)
Aβ42/40	80 (55.2)	919 (55.9)

*Note:* Data are mean (SD), median (IQR), or *n* (%) unless otherwise indicated.

Abbreviations: AD, Alzheimer's disease; Aβ, amyloid‐β; GFAP, glial fibrillary acidic protein; IQR, interquartile range; MMSE, Mini‐Mental State Examination; MSD, Meso Scale Discovery; PET, positron emission tomography; SUVR, standardized uptake value ratio.

^a^ Percentages are calculated among participants with available APOE genotype.

### 3.2 Full Model Performance

In ADNI, the complete GRAD model achieved an AUC of 0.915 (95% CI, 0.859–0.961), accuracy of 87.6% (95% CI, 82.1%–93.1%), 89.4% sensitivity (95% CI, 82.7%–95.6%), 85.0% specificity (95% CI, 75.0%–93.0%), PPV 89.4%, NPV 85.0%, LR+ 5.96, LR‐ 0.12, and a Brier score of 0.108 (Table S5). The model correctly classified 127 of 145 participants, with 76 true positives and 51 true negatives. Performance was consistent across cognitive status (MCI 0.906, 95% CI 0.839–0.958; AD dementia 0.958, 0.861–1.000), APOE ε4 status (ε4–0.921, 0.851–0.979; ε4+ 0.817, 0.624–0.978), sex (male 0.940, 0.882–0.983; female 0.865, 0.726–0.974), and age tertiles (0.853–0.969) (Figure S1). At the 90% sensitivity operating threshold (*p* < 0.451), the model achieved 91.8% sensitivity with 81.7% specificity (NPV 87.5%, LR‐ 0.10); at the 90% specificity threshold (*p* > 0.667), 90.0% specificity with 81.2% sensitivity (PPV 92.0%, LR+ 8.12) (Table S6).

In A4 and LEARN, the ADNI‐trained model achieved AUC 0.879 (95% CI, 0.861–0.895), with 76.7% sensitivity, 88.6% specificity, PPV 93.9%, and NPV 62.3%. Subgroup performance in the external cohort was similarly stable (Figure 3A). Individual‐level predicted probabilities correlated with Aβ‐PET centiloid values (Spearman *ρ* = 0.748, *p* < 0.001), indicating that model outputs track continuous amyloid burden beyond binary classification. Across the full pipeline, 89.0% of ADNI and 90.7% of A4 participants received a definitive Aβ classification without imaging.

While discrimination and routing transferred, calibration, in large, did not. The calibration slope was 1.04 and the intercept +0.93 with mean predicted risk 57.5% against an observed prevalence of 69.6% (Brier 0.145) (Figure 3D).

### 3.3 Stage 1 Performance

In ADNI, using only the Gatekeeper model, 96 of the 145 participants (66.2%) placed outside the gray zone and were resolved with an AUC of 0.939 (95% CI, 0.866–0.995). Thirty seven cases were classified as Aβ negative (NPV 91.9%), 59 cases were classified as Aβ positive (PPV 94.9%), for an accuracy of 93.8% among resolved cases, and 49 cases were in the gray zone (33.8%) which were routed to Stage 2.

In A4 and LEARN, the same model resolved 1014 of 1644 participants (61.7%) with an AUC of 0.904, classifying 408 as Aβ negative (NPV 70.1%) and 606 as Aβ positive (PPV 97.0%), for an accuracy of 86.2% among resolved cases, and 630 (38.3%) entered the gray zone.

The fitted Stage 1 model was logit(P) = −1.023 + 1.124 × *p*‐Tau217_Z, so the routing thresholds correspond to fixed harmonized p‐Tau217 values and can be applied to a new patient by hand.

### 3.4 Stage 2 Performance

In ADNI, within the gray zone (*n* = 49) (Figure 2A), Stage 2 achieved an AUC of 0.781 (95% CI, 0.630–0.913) with 75.5% accuracy, 76.9% sensitivity, and 73.9% specificity, against 0.739 (0.592–0.867) for p‐Tau217 alone on the same participants (+0.042; DeLong *p* = 0.54) (Figure 2B). In A4 and LEARN, within the gray zone (*n* = 630) (Figure 2A), Stage 2 achieved an AUC of 0.793 (95% CI, 0.753–0.832) with 70.8% accuracy, 66.7% sensitivity and 80.0% specificity (Figure 2C), against 0.719 (0.675–0.762) for p‐Tau217 alone (+0.073)—both comparisons are reported in full in Section 3.5.

**FIGURE 2:**
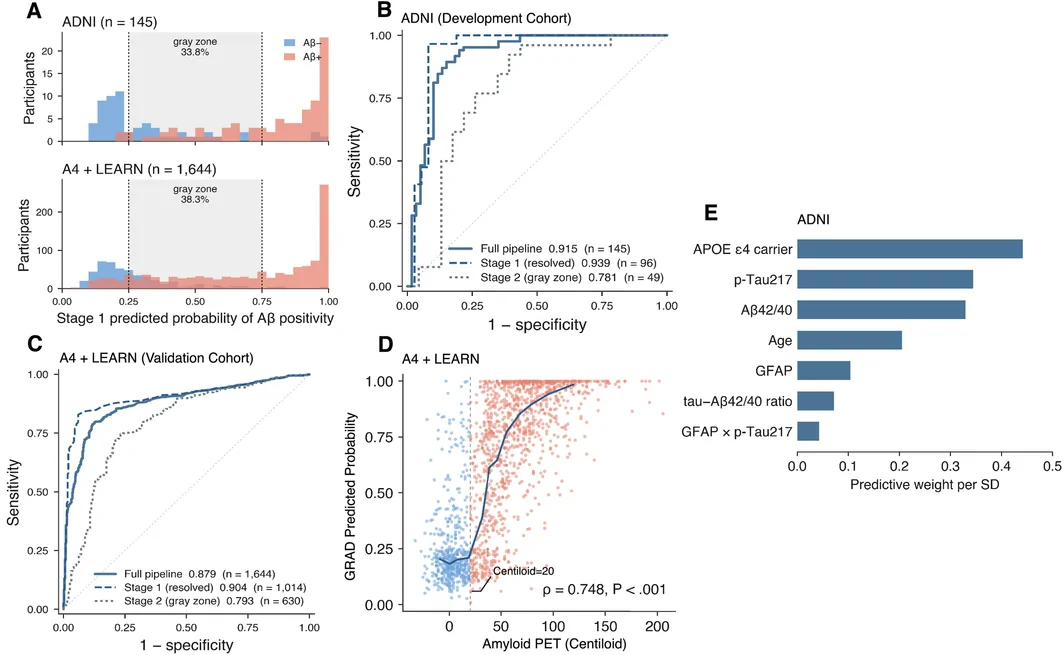
Model cross‐cohort performance. (A) Distribution of Stage 1 predicted probability of Aβ positivity in ADNI (top, *n* = 145) and A4 + LEARN (bottom, *n* = 1644), stratified by Aβ‐PET status. Shading marks the 0.25–0.75 gray zone, which contained 33.8% of ADNI and 38.3% of A4 + LEARN participants. (B) Receiver operating characteristic curves in the ADNI development cohort under leave‐one‐out cross‐validation: Full pipeline (AUC 0.915, *n* = 145), Stage 1 among resolved cases (0.939, *n* = 96), and Stage 2 within the gray zone (0.781, *n* = 49). (C) The same curves in the A4 + LEARN validation cohort, scored once by the ADNI‐fitted model without refitting: Full pipeline (0.879, *n* = 1644), Stage 1 (0.904, *n* = 1014), Stage 2 (0.793, *n* = 630). (D) GRAD predicted probability against Aβ‐PET Centiloid value in A4 + LEARN, colored by Aβ‐PET status; the dashed line marks the Centiloid 20 positivity threshold and the solid line is a running median. (E) Ranked predictive weights of each feature and interaction term in Reflex (Stage 2) of ADNI.

We found that discrimination inside the band rests mainly on three predictors: APOE ε4 carrier status (odds ratio per SD 3.14; 95% CI, 1.53–6.44; *p* = 0.002), p‐Tau217 (2.34; 1.20–4.56; *p* = 0.013) and Aβ42/40 (0.32; 0.13–0.77; *p* = 0.011). GFAP (0.80; *p* = 0.48) and the GFAP by p‐Tau217 interaction (0.94; *p* = 0.83) contribute little (Figure 2E; Table S7). Drop‐one ablation in the external cohort gives the same ordering: removing APOE ε4 costs 0.034 AUC and Aβ42/40 costs 0.030, while removing GFAP, age or the interaction did not significantly degrade performance (Table S8).

Stage 2 resolved 33 of the 49 ADNI gray zone cases (67.3%) and 477 of the 630 in A4 (75.7%), leaving 16 (11.0% of the development cohort) and 153 (9.3% of the validation cohort) indeterminate and referred for confirmatory Aβ‐PET.

### 3.5 Benchmarking Against p‐Tau217 and p‐Tau181

Against p‐Tau217 alone, Stage 2 improved gray zone discrimination by 0.042 in ADNI (0.739 to 0.781; DeLong *p* = 0.54, *n* = 49) and by 0.073 in A4 (0.719 to 0.793; DeLong *p* < 0.001, *n* = 630). Applying the identical 0.40–0.60 indeterminate band to both scores in the external cohort, p‐Tau217 alone resolved 41.4% of gray zone patients correctly, resolved 22.7% wrongly, and left 35.9% needing PET; GRAD resolved 56.2% correctly, 19.5% wrongly, and left 24.3% needing PET (Figure 3B).

**FIGURE 3:**
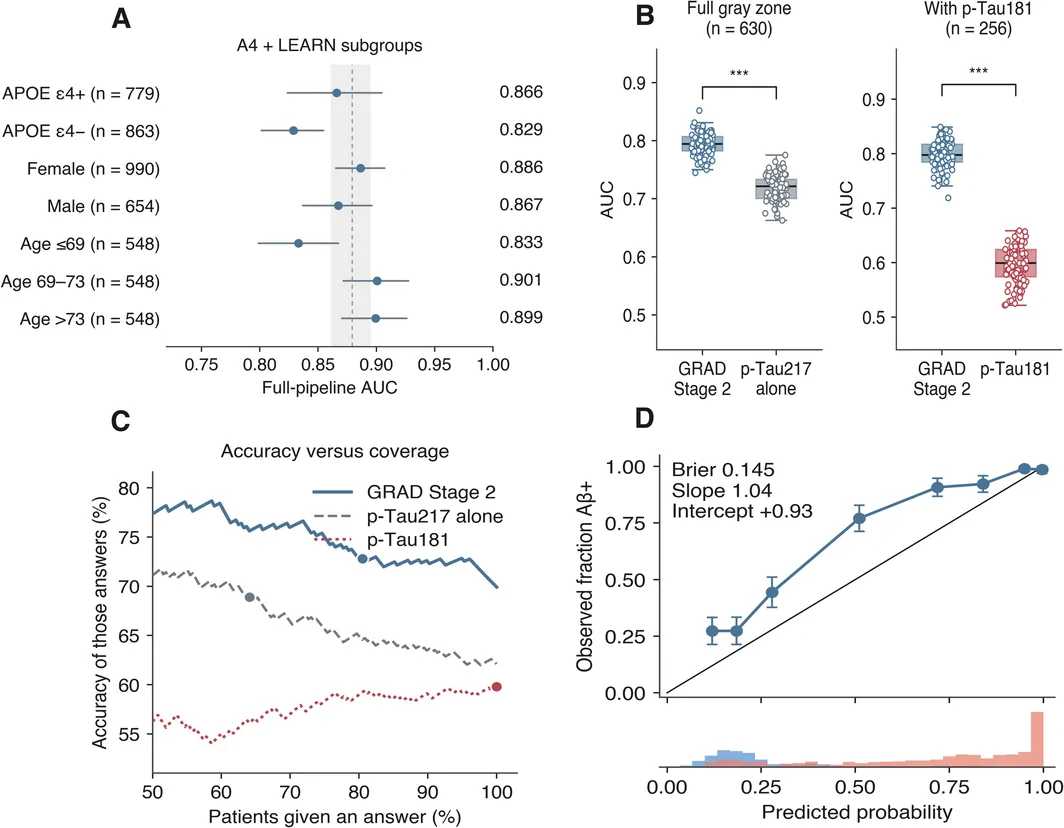
External validation and benchmarking analysis. (A) Full‐pipeline AUC across clinical subgroups of the A4 + LEARN validation cohort, with 95% bootstrap confidence intervals; the dashed line and shaded band show the whole‐cohort estimate. (B) Bootstrap distributions of gray zone AUC. Left: GRAD Stage 2 versus p‐Tau217 alone across the full external gray zone. Right: GRAD Stage 2 versus p‐Tau181 in the subset with a visit‐matched p‐Tau181 measurement. Boxes show the interquartile range, the horizontal line the median. ***p < 0.001. (C) Accuracy against coverage in the external gray zone. Note as the low‐confidence band is widened and fewer patients are given an answer, accuracy among those answered rises. GRAD Stage 2 and p‐Tau217 alone under the 0.40–0.60 indeterminate band, and p‐Tau181 at 100% coverage (Youden cutoff). (D) Calibration of the full pipeline in A4 + LEARN. Observed versus predicted Aβ positivity by decile of predicted probability with 95% confidence intervals; diagonal is perfect calibration. The lowest panel is the distribution of predicted probabilities by Aβ‐PET status.

The p‐Tau181 benchmark was restricted to the 256 A4 gray zone participants with a visit‐matched p‐Tau181 measurement (55.5% Aβ‐positive). In this subset, GRAD Stage 2 achieved an AUC of 0.799 (95% CI, 0.740–0.853), against 0.597 (95% CI, 0.526–0.668) for p‐Tau181 (DeLong *p* < 0.001) and 0.743 (95% CI, 0.677–0.802) for p‐Tau217 alone (*p* = 0.06). GRAD's AUC here (0.799) differs marginally from its AUC across the full external gray zone (0.793, *n* = 630) because this analysis is confined to the p‐Tau181 subset; the two are not competing estimates of the same quantity.

Giacomucci et al. report that p‐Tau181 correctly reclassified 77.4% (24 of 31) of their indeterminate cases [17]. Applying the same rule—a single dichotomous cutoff derived by Youden's index, in A4, p‐Tau181 classified 59.8% of our gray zone participants correctly and 40.2% incorrectly. GRAD, evaluated on the same 256 participants, classified 58.6% correctly and 21.9% incorrectly while referring the remaining 19.5% for Aβ‐PET (Table 2, Figure 3B,C).

**TABLE 2:** Benchmarking GRAD Stage 2 against p‐Tau217 alone and p‐Tau181 within the gray zone.

Cohort and score	*n*	AUC (95% CI)	Δ AUC	Resolved correctly, %	Resolved incorrectly, %	Referred for Aβ‐PET, %
ADNI gray zone						
p‐Tau217 alone	49	0.739 (0.592–0.867)	+0.042	49.0	14.3	36.7
GRAD Stage 2	49	0.781 (0.630–0.913)	—	57.1	10.2	32.7
A4 + LEARN gray zone						
p‐Tau217 alone	630	0.719 (0.675–0.762)	+0.073	41.4	22.7	35.9
GRAD Stage 2	630	0.793 (0.753–0.832)	—	56.2	19.5	24.3
p‐Tau181 (Youden cutoff)	256	0.597 (0.526–0.668)	+0.202	59.8	40.2	0
p‐Tau217 alone	256	0.743 (0.677–0.802)	+0.057	44.1	19.9	35.9
GRAD Stage 2	256	0.799 (0.740–0.853)	—	58.6	21.9	19.5

*Note:* Each block reports one comparison. GRAD Stage 2 is the reference within each block, so Δ AUC and the accompanying *p*‐value are GRAD minus that comparator by DeLong test. p‐Tau181 was applied as a single dichotomous cutoff derived by Youden index, following the original integration study. The p‐Tau181 subset is 256 A4 + LEARN gray zone participants with a visit‐matched p‐Tau181 measurement (55.5% Aβ‐positive).

Abbreviations: Aβ, amyloid‐β; AUC, area under the receiver operating characteristic curve; CI, confidence interval; PET, positron emission tomography. Other abbreviations as in Table 1.

### 3.6 Cost Impact Simulation

Using the external cohort's routing rates, Universal PET cost $30,000,000 (10,000 scans), p‐Tau217 screening with PET for every gray zone case cost $15,000,000 (3832 scans), a relative 50% saving, and the GRAD staged algorithm cost $7,250,000 (931 scans), a relative 76% saving, or $725 per patient. Using the development cohort's routing rates, the corresponding GRAD figure was $7,655,000 (1103 scans; 74% saving; $766 per patient). Under development‐cohort routing, p‐Tau217 screening with PET for every gray zone case cost $13,638,000 (3379 scans; 55% saving). Measured against this p‐Tau217‐plus‐PET workflow rather than universal imaging, GRAD was 44% less costly under development‐cohort routing and 52% less costly under validation‐cohort routing. Across both cohorts, GRAD reduced PET utilization by 89%–91%.

When assessing simulation results as cost per correct diagnosis—counting PET‐referred individuals as correctly diagnosed—the external cohort cost was $3000 for universal PET, $1639 for p‐Tau217 screening with PET for the gray zone, and $863 for GRAD. In the development cohort this was $3000, $1423, and $828, respectively (Figure 4).

**FIGURE 4:**
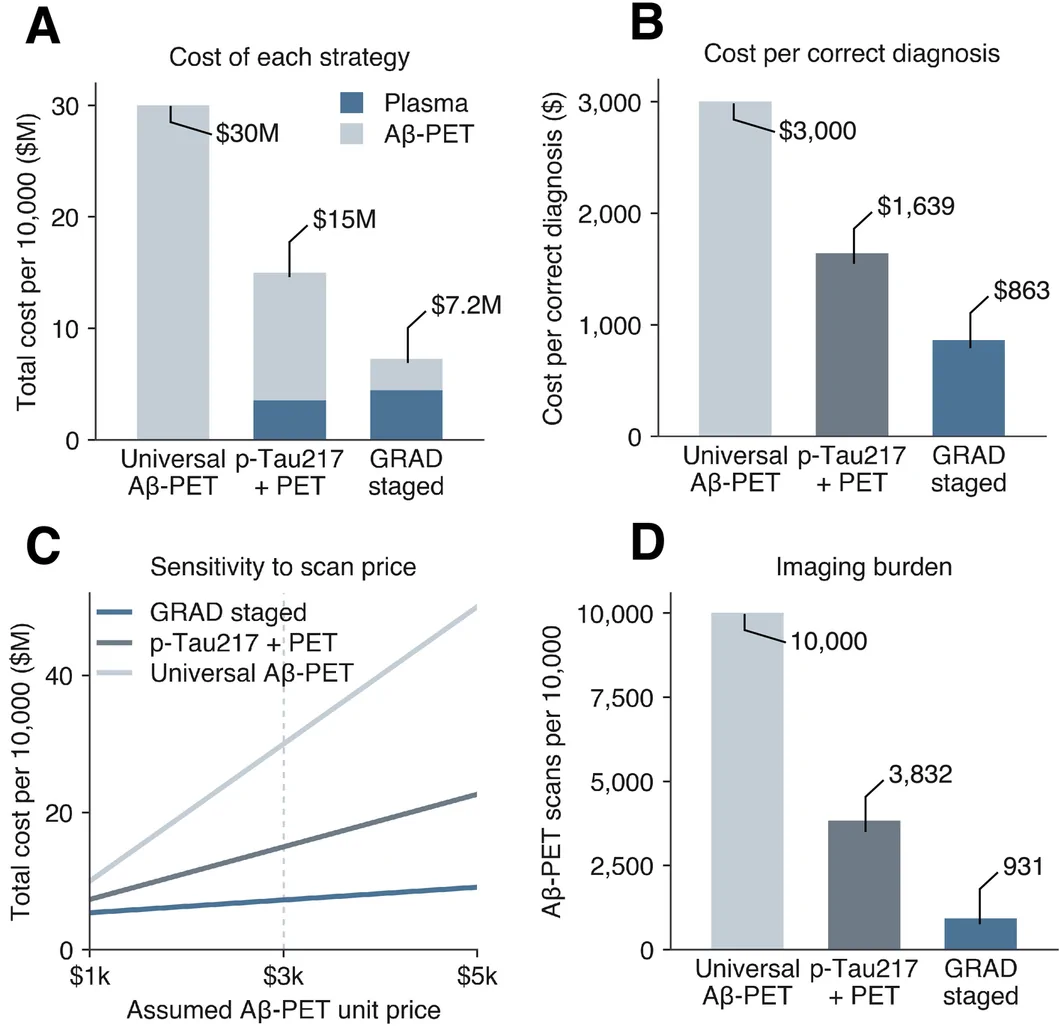
Health economic savings simulation. All panels show a hypothetical cohort of 10,000 patients using external‐cohort (A4 + LEARN) routing rates. The unit costs are as follows: $350 per p‐Tau217 test, $250 for the Stage 2 multianalyte panel, and $3000 per Aβ‐PET scan. (A) Total cost of each diagnostic strategy, partitioned into plasma assay and the respective Aβ‐PET components. (B) Cost per correct diagnosis, counting PET‐referred patients as correctly diagnosed. (C) Sensitivity of total cost to the assumed Aβ‐PET unit price across $1000–$5000. (D) Imaging burden per 10,000 patients, showcasing number of scans per group.

## 4 Discussion

In this multicohort study, we developed and externally validated a two‐stage plasma algorithm designed to improve Aβ classification when p‐Tau217 is indeterminate. The principal finding was that additional biomarker and genetic information provided meaningful discrimination within this difficult subgroup and transported across two clinically distinct research cohorts. In the A4/LEARN external validation cohort, GRAD Stage 2 improved gray‐zone AUC from 0.719 with p‐Tau217 alone to 0.793 (*p* < 0.001), increased the proportion of participants correctly classified without imaging from 41.4% to 56.2%, and reduced the proportion requiring Aβ‐PET from 35.9% to 24.3%. These findings support a staged approach in which p‐Tau217 resolves high‐confidence cases, additional plasma and genetic information is used selectively for indeterminate results, and confirmatory imaging is retained for residual uncertainty.

Our results also confirm that a substantial p‐Tau217 gray zone is present across clinically distinct portions of the AD continuum. A two‐cutoff p‐Tau217 strategy resolved 96 of 145 cognitively impaired ADNI participants (66.2%) and 1014 of 1644 cognitively unimpaired A4/LEARN participants (61.7%), with accuracies of 93.8% and 86.2%, respectively. The remaining gray zone comprised 33.8% of ADNI and 38.3% of A4/LEARN participants, consistent with prior studies reporting that approximately 30%–50% of tested individuals fall within an indeterminate range [5, 14, 15, 16]. Rather than replacing p‐Tau217, GRAD is therefore intended to complement it by concentrating additional testing on the subset for whom the initial biomarker provides insufficient certainty. Accordingly, GRAD should be interpreted as a decision‐support and triage strategy for determining who may be classified from blood‐based testing and who still requires confirmatory testing, rather than as a substitute for Aβ‐PET in all individuals.

Importantly, the principal evidence for GRAD's incremental value came from external validation. Although Stage 2 improved gray‐zone AUC over p‐Tau217 alone by 0.042 in ADNI, this difference was not statistically significant in the small development gray zone (*n* = 49; *p* = 0.54). In A4/LEARN, however, the improvement was larger (+0.073 AUC; *p* < 0.001) despite application to a clinically distinct population, use of a different p‐Tau217 assay platform, and scoring without model refitting. GRAD also performed favorably against p‐Tau181 integration in the subset with visit‐matched measurements, achieving an AUC of 0.799 compared with 0.597 for p‐Tau181 (*p* < 0.001). Model discrimination was broadly consistent across sex subgroups in both cohorts (Figure S1), although these subgroup analyses were descriptive and were not designed to establish equivalence of performance between males and females. Together, these findings suggest that the incremental information provided by the Reflex stage is not confined to the development sample and may offer greater utility for resolving indeterminate p‐Tau217 results than reliance on an additional tau marker alone.

Within the Reflex stage, the strongest contributors to gray‐zone discrimination were APOE ε4 carrier status, p‐Tau217, and the Aβ42/40 ratio. In contrast, GFAP and its interaction with p‐Tau217 contributed little additional discrimination, and external ablation analyses similarly showed the largest reductions in AUC when APOE ε4 or Aβ42/40 was removed. This pattern suggests that, once p‐Tau217 has identified an indeterminate subgroup, complementary information from amyloid‐related measures and genetic risk may be more informative than additional astroglial markers. More broadly, these findings support the rationale for a reflex strategy that combines biologically complementary signals rather than adding biomarkers from the same pathological axis.

The Alzheimer's Association Clinical Practice Guideline recommends ≥ 90% sensitivity with ≥ 75% specificity for a blood‐based biomarker used as a triage test, and ≥ 90% sensitivity and specificity when used in place of Aβ‐PET [3]. In the ADNI development cohort, GRAD could be operated at thresholds consistent with the recommended triage benchmark: at the rule‐out threshold, sensitivity was 91.8% with 81.7% specificity (NPV 87.5%), while at the rule‐in threshold, specificity was 90.0% with 81.2% sensitivity (PPV 92.0%). These operating points were derived in the development cohort; however, they require prospective validation before fixed thresholds can be assumed to meet these benchmarks in other clinical populations. In external validation, the Reflex stage evaluated in isolation achieved 66.7% sensitivity and 80.0% specificity, below the performance recommended for replacement of Aβ‐PET. Together, these findings support GRAD as a staged triage and trial‐enrichment approach that can reduce reliance on confirmatory imaging while retaining Aβ‐PET for residual uncertainty, rather than as a standalone replacement for confirmatory imaging.

The clinical utility of GRAD will depend on the population tested and the respective consequences of false‐positive and false‐negative classifications. Because predictive values vary with amyloid prevalence, the same operating characteristics yield different projected performance across settings. For example, at an amyloid prevalence of approximately 20%, a negative GRAD result would have a projected NPV of 93.8%, suggesting greatest potential value as a rule‐out strategy [7, 47]. At 50% prevalence, projected PPV and NPV are 87.0% and 79.2%, respectively, whereas at 70% prevalence projected PPV increases to 94.0% (Table S9). These estimates illustrate the importance of clinical context but should not be interpreted as prospectively validated performance in primary care, memory clinics, or prevention trials. Thresholds may ultimately require setting‐specific calibration depending on amyloid prevalence and the relative consequences of false‐positive and false‐negative classifications. Furthermore, future model designs incorporating novel markers, such as %p‐Tau217, MTBR‐tau243 or brain‐derived tau [48, 49, 50], may further distinguish GRAD‐classified Aβ+ individuals.

A potential implementation strategy would keep p‐Tau217 as an initial screen, apply the Reflex multi‐analyte panel to individuals with indeterminate results, and then reserve confirmatory Aβ‐PET for uncertain cases after Stage 2. In our cohorts, these residual indeterminate cases represented 11.0% of the ADNI development cohort and 9.3% of the A4/LEARN validation cohort. This staged framework could therefore reduce reliance on Aβ‐PET while preserving confirmatory imaging for residual uncertainty. Prospective validation in clinically representative populations, including assessment of setting‐specific calibration and thresholds, is needed before GRAD is used to guide individual clinical or trial‐enrollment decisions.

Utilizing GRAD's diagnostic strategy carries potential economic and access implications. In our simulation, GRAD reduced projected testing costs by 74%–76% relative to universal PET and by 44%–52% relative to p‐Tau217 screening with PET confirmation of the gray zone, with estimated costs of $725–766 per patient. GRAD also had the lowest modeled cost per correct diagnosis ($828–863), although these estimates do not incorporate the downstream consequences or costs of misclassification. Blood‐based testing generally requires less specialized infrastructure than Aβ‐PET, although the multi‐analyte panel required for GRAD's Reflex stage is not yet routinely available in all settings. By reducing projected PET requirements by 89%–91%, this staged approach could be particularly useful in settings where access to specialized neuroimaging is limited, including rural and underserved communities where dementia diagnoses are often less timely and complete [51, 52].

### 4.1 Limitations

Our study also has several limitations. First, both cohorts are predominantly white (89% of ADNI and 94% of A4) and highly educated, so GRAD's performance in racially and ethnically diverse populations is unknown and may differ [53, 54]. The development and validation cohorts were also heterogeneous in several important respects, including cognitive status, sex distribution, Aβ prevalence, p‐Tau217 assay platform, and definition of Aβ positivity. These differences provide a stringent test of model transportability but also complicate direct comparison of performance across cohorts and may contribute to observed differences in predictive values and calibration. Although discrimination and routing performance transferred to the external cohort, further validation is needed in independent clinical populations using locally applicable assays and thresholds before clinical implementation. Second, our study did not account for renal function, a known confounder of plasma biomarker concentrations [55, 56, 57], or systematically characterize comorbid proteinopathies [4, 20] and mixed pathologies [33]. Such comorbidities could alter biomarker concentrations or the relationship between plasma biomarkers and Aβ‐PET, and GRAD therefore requires validation in more clinically heterogeneous populations with common medical and neurological comorbidities. Third, our development cohort is small, and its gray zone contains only 49 participants. The improvement in ADNI Stage 2 did not reach significance on its own (*p* = 0.54)—we therefore rest GRAD's benefit on the well‐powered external cohort; the internal estimate is reported as supportive. Fourth, our Stage 2 required a multianalyte panel that is not yet routine in all settings. Analyte availability was also uneven: Aβ42/40 was measured in 919 of 1644 A4/LEARN participants, and missing values were imputed from training‐set medians. Although results were consistent in the complete‐panel sensitivity analysis without imputation (Table S2), missingness may not have occurred randomly, and imputation cannot fully recover information from unmeasured biomarkers. The analysis benchmarking GRAD against p‐Tau181 also carries limitations. It was possible only in A4, because ADNI's plasma p‐Tau181 was assayed a median of 4 years before the p‐Tau217 draws used here and only 7 of 49 ADNI gray zone participants have a visit‐matched value. We suggest the comparison should be read as a single‐cohort comparison between populations that differ (impaired versus unimpaired). Fifth, our cross‐sectional design evaluates GRAD for classification of contemporaneous Aβ status and does not assess longitudinal changes in biomarker concentrations, prediction of future Aβ accumulation or cognitive decline, or performance during treatment monitoring. Longitudinal validation will therefore be necessary to determine whether GRAD probabilities and classification thresholds remain stable over time and whether the framework has utility for repeated testing. Sixth, although discrimination transferred across cohorts, calibration did not fully transport, indicating that predicted probabilities and fixed probability thresholds may require recalibration before application in populations with different amyloid prevalence or assay characteristics. Finally, our cost simulation projects savings hypothetically rather than with real‐world data. It ignores geographic variation, and does not price the cost of misclassification, including ARIA. Real cohort financial data would be required to demonstrate the economic case directly.

## 5 Conclusion

As p‐Tau217 is increasingly incorporated into early AD screening, GRAD offers a staged approach for addressing the substantial subgroup with indeterminate results. In external validation, the Reflex stage improved discrimination over p‐Tau217 alone within the gray zone while retaining Aβ‐PET for residual uncertainty. Across the full pipeline, 89.0% of ADNI and 90.7% of A4/LEARN participants received an Aβ classification without imaging, suggesting that selective reflex testing could substantially reduce reliance on confirmatory PET. Modeled testing costs were also 44%–52% lower than strategies relying on PET for all indeterminate p‐Tau217 results, although these projections require confirmation with real‐world economic data. At present, GRAD should be viewed as a potential triage and trial‐enrichment strategy rather than a replacement for confirmatory imaging. Future work should prospectively evaluate the algorithm in clinically heterogeneous and racially and ethnically diverse populations, assess setting‐specific calibration and thresholds, and determine whether the projected reductions in PET use and testing costs are realized in clinical practice.

## Author Contributions

**Harthik Parankusham:** conceptualization, methodology, software, formal analysis, investigation, data curation, writing – original draft, project administration. **Casey Vanderlip:** validation, writing – review and editing. **Colin Birkenbihl:** methodology, supervision, review and editing. **Eashwar Krishna:** formal analysis, investigation, data curation. **Chizobam Ugboaja:** visualization, writing – original draft. **Andrew E. Budson:** supervision, resources, writing – review and editing. **Brandon Frank:** conceptualization, supervision, project administration, writing – review and editing.

## Funding

A.E.B. is funded in part by the Veterans Health Administration (I01 CX002400) and NIH/NIA (P30 AG072978). B.F. is funded in part by the Veterans Health Administration (IK2 CX002625) and NIH/NIA (P30 AG072978).

## Ethics Statement

ADNI was approved by the institutional review boards of all participating institutions, and the A4 and LEARN Studies were approved by the institutional review board at each participating site. All participants provided written informed consent prior to enrollment. Our study was not prospectively registered.

## Conflicts of Interest

The authors declare no conflicts of interest.

## Supporting information


**Table S1:** Analyte availability by cohort and assay platform. Counts are participants with a nonmissing value for each analyte. ADNI p‐Tau217 was measured on the Lumipulse G assay (Fujirebio, University of Pennsylvania) and on the Janssen assay; the Janssen subset contributed p‐Tau217 only. A4 + LEARN p‐Tau217 was measured on the Lilly Research Laboratories MSD immunoassay, with GFAP and Aβ42/40 on the Roche Elecsys platform in a subset of the cohort. ADNI counts refer to the 145 cognitively impaired participants who formed the development cohort. Because the two cohorts used different p‐Tau217 platforms, all plasma biomarkers were reference‐anchored within cohort and platform before analysis (Section 2.3).
**Table S2:** Complete‐panel sensitivity analysis in A4 + LEARN. Participants are those in the external validation cohort with a non‐missing value for all seven Stage 2 features. The ADNI‐fitted model is unchanged; only the participants scored differ. Panel availability was informative: Aβ prevalence was 55.7% among complete‐panel participants against 87.1% among those requiring imputation, so the two subgroups are not interchangeable and the difference in AUC between them (−0.034; 95% CI, −0.075 to +0.008) reflects case mix as well as any effect of imputation. The interval does not exclude a difference of clinical relevance and should not be read as demonstrating equivalence.
**Table S3:** Sensitivity of external validation to the reference‐anchoring definition. The ADNI‐fitted model is held completely fixed; only the mu and sigma used to reference‐anchor the validation cohort change. Stage 1 AUC is computed over the whole cohort and is identical to four decimal places under every definition, because the Stage 1 probability is a monotone function of raw p‐Tau217 whatever reference is chosen. The final row draws the reference from a random half of the Aβ‐negative participants, repeated 200 times, and reports the median with the 2.5th to 97.5th percentile range. The two whole‐cohort definitions deliberately mis‐anchor the scale, since 69.6% of the cohort is Aβ‐positive, and are included to bound the worst case: discrimination is preserved, while the 0.5 probability threshold is displaced and accuracy falls accordingly. AUC, area under the receiver operating characteristic curve.
**Table S4:** Cost model unit costs and assumptions. Unit costs were drawn from 2024 U.S. Medicare clinical laboratory and physician fee schedules (references 43–45). Where a range was plausible we adopted the higher plasma figure and a conservative Aβ‐PET price so that savings are not overstated. Costs are undiscounted, expressed in 2024 U.S. dollars, and exclude clinician time, downstream treatment, and the consequences of misclassification.
**Table S5:** Diagnostic performance of GRAD by stage and cohort. Stage 1 rows cover only the participants that stage resolves; its sensitivity and specificity use the 0.25 and 0.75 routing thresholds as the decision rule. Stage 2 rows cover the gray zone. Stage 2 and full‐pipeline values use a 0.5 probability threshold. Confidence intervals are percentile bootstrap over participants (2000 resamples). ADNI values are leave‐one‐out cross‐validated; A4 + LEARN values are from the ADNI‐fitted model applied without refitting. AUC, area under the receiver operating characteristic curve; LR, likelihood ratio; NPV, negative predictive value; PPV, positive predictive value.
**Table S6:** Operating points in the ADNI development cohort. Operating points are derived from the ADNI development cohort (*n* = 145) through leave‐one‐out cross‐validated predictions of the full pipeline. We show operating points over the range from which the model can be tuned. They have not been validated prospectively and should not be adopted as fixed thresholds in other populations without recalibration. LR, likelihood ratio; NPV, negative predictive value; PPV, positive predictive value.
**Table S7:** Stage 2 associations and model coefficients. All estimates are from the ADNI gray zone (*n* = 49). Univariable odds ratios fit each feature alone and are the values quoted in Section 3.4. Multivariable odds ratios fit all seven features together without penalization and are reported so that the two can be compared directly; their intervals are wide because the features are correlated and the sample is small. The final column gives the L2‐penalized coefficients of the deployed Stage 2 model on standardized features, whose magnitudes are plotted in Figure 2E. The negative coefficient for Aβ42/40 reflects that a lower ratio indicates greater amyloid burden. The univariable estimates and the penalized coefficients agree in ranking APOE ε4, p‐Tau217 and Aβ42/40 highest. The unpenalized multivariable estimates are unstable, with p‐Tau217 and the tau−Aβ42/40 ratio taking large coefficients of opposing sign because the two are collinear by construction; this instability is why the deployed model is L2‐penalized. OR, odds ratio.
**Table S8:** Feature ablation in the A4 + LEARN gray zone. Each row refits Stage 2 on the ADNI gray zone with the stated feature set and scores the A4 + LEARN gray zone (*n* = 630) without refitting. A negative Δ AUC indicates that removing the feature degraded external discrimination.
**Table S9:** Predictive values across assumed Aβ prevalence. Positive and negative predictive value depend on how common Aβ positivity is in the population being tested, whereas sensitivity and specificity do not. Values are derived from the full‐pipeline operating point in A4 + LEARN (76.7% sensitivity, 88.6% specificity) applied at each assumed prevalence by Bayes theorem. The 30% and 50% rows approximate a primary care memory complaint population and a specialist memory clinic respectively; the observed prevalence was 69.6% in A4 + LEARN and 58.6% in ADNI. NPV, negative predictive value; PPV, positive predictive value.
**Figure S1:** Full‐pipeline discrimination by subgroup, ADNI development cohort. Area under the receiver operating characteristic curve for the complete GRAD pipeline within clinical subgroups of the ADNI development cohort (*n* = 145), with 95% percentile bootstrap confidence intervals. The dashed line and shaded band show the whole‐cohort estimate and its interval. Intervals are wide for the smaller strata, notably AD dementia (*n* = 28) and APOE ε4 carriers (*n* = 61), and should be read as imprecision rather than instability. The corresponding analysis in the A4 + LEARN validation cohort is Figure 3A; cognitive status cannot be stratified there because all participants are cognitively unimpaired.

## Data Availability

All code for our analysis is available at: https://github.com/hsparankusham/GRAD‐grayzone‐classifier. The ADNI datasets analyzed during this study are available from the ADNI repository at https://adni.loni.usc.edu, and the A4/LEARN datasets are available through the Alzheimer's Clinical Trial Consortium's Global Research & Imaging Platform at https://www.actcinfo.org, both subject to registered data use agreements.
