# Supplementary material for: A Two‐Stage Plasma Biomarker Algorithm Reduces Amyloid PET Use and the p‐Tau217 Gray Zone
Source: Ann Clin Transl Neurol. 2026 Oct 19:10.1002/acn3.70560. Online ahead of print. doi: 10.1002/acn3.70560 (PMC13680551; doi:10.1002/acn3.70560)
Supplement: Supplementary file 1 [file ACN3-9999-0-s001.pdf]

ANNALS
of Clinical and Translational Neurology

Supplementary Materials for

A Two-Stage Plasma Biomarker Algorithm Reduces Amyloid PET Use and the p-Tau217 Gray Zone

Harthik Parankusham[1*], Casey Vanderlip B.S.[2], Colin Birkenbihl B.S., M.S., Ph.D.[3], Eashwar Krishna B.S.[4], Chizobam Ugboaja B.S.[5], Andrew E. Budson M.D.[6], Brandon Frank Ph.D.[6] and for the Alzheimer's Disease Neuroimaging Initiative†

*Corresponding author: harthik.parankusham@uconn.edu (H.P)

†**Alzheimer's Disease Neuroimaging Initiative:** Data used in the preparation of this article were obtained from the Alzheimer's Disease Neuroimaging Initiative (ADNI) database (adni.loni.usc.edu).

The PDF file includes:

Tables S1 to S9
Figure S1

Supplementary Table S1. Analyte availability by cohort and assay platform.

Cohort and assay platform	n	p-Tau217	GFAP	Aβ42/40	APOE genotype
ADNI - Janssen immunoassay	64	64	0	0	64
ADNI - Lumipulse G (Fujirebio, UPENN)	81	81	81	80	68
ADNI - all	145	145	81	80	132
A4 + LEARN - Lilly MSD / Roche Elecsys	1,644	1,644	931	919	1,642

Counts are participants with a non-missing value for each analyte. ADNI p-Tau217 was measured on the Lumipulse G assay (Fujirebio, University of Pennsylvania) and on the Janssen assay; the Janssen subset contributed p-Tau217 only. A4 + LEARN p-Tau217 was measured on the Lilly Research Laboratories MSD immunoassay, with GFAP and Aβ42/40 on the Roche Elecsys platform in a subset of the cohort. ADNI counts refer to the 145 cognitively impaired participants who formed the development cohort. Because the two cohorts used different p-Tau217 platforms, all plasma biomarkers were reference-anchored within the cohort and platform before analysis (Section 2.3).

Supplementary Table S2. Complete-panel sensitivity analysis in A4 + LEARN.

A4 + LEARN subgroup	n	Aβ+, %	Full pipeline AUC (95% CI)	Gray zone AUC	Accuracy, %
All participants	1,644	69.6	0.879 (0.861-0.895)	0.793	80.3
Complete panel	915	55.7	0.854 (0.828-0.878)	0.754	79.3
Imputed ≥1 analyte	729	87.1	0.888 (0.852-0.918)	0.765	81.5

Participants are those in the external validation cohort with a non-missing value for all seven Stage 2 features. The ADNI-fitted model is unchanged; only the participants scored differ. Panel availability was informative: Aβ prevalence was 55.7% among complete-panel participants against 87.1% among those requiring imputation, so the two subgroups are not interchangeable and the difference in AUC between them (-0.034; 95% CI, -0.075 to +0.008) reflects case mix as well as any effect of imputation. The interval does not exclude a difference of clinical relevance and should not be read as demonstrating equivalence.

Supplementary Table S3. Sensitivity of external validation to the reference-anchoring definition.

A4 + LEARN reference definition	Uses Aβ status	Stage 1 AUC	Gray zone, %	Gray zone AUC	Full pipeline AUC	Accuracy, %
Aβ-negative participants (primary)	Yes	0.867	38.3	0.793	0.879	80.3
Lowest p-Tau217 tercile	No	0.867	21.2	0.708	0.874	82.4
Whole cohort, median and IQR	No	0.867	42.1	0.787	0.871	66.3
Whole cohort, mean and SD	No	0.867	34.4	0.748	0.867	58.2
Random half of Aβ-negative participants	Yes	0.867	37.2 (31.6-42.5)	0.790 (0.777-0.808)	0.880 (0.878-0.881)	80.4 (78.8-82.2)

Because our reference population was defined by Aβ-status, the transformation we applied to the validation cohort was estimated using that cohort's outcome labels. We thus repeated the external validation with the A4 reference redefined, without reference to Aβ-status, as the lowest tertile of the p-Tau217 distribution (n=548, 33.9% of whom were actually Aβ-positive) (Table S3).

The ADNI-fitted model is held completely fixed; only the mu and sigma used to reference-anchor the validation cohort change. Stage 1 AUC is computed over the whole cohort

and is identical to four decimal places under every definition, because the Stage 1 probability is a monotone function of raw p-Tau217 whatever reference is chosen. The final row draws the reference from a random half of the Aβ-negative participants, repeated 200 times, and reports the median with the 2.5th to 97.5th percentile range. The two whole-cohort definitions deliberately mis-anchor the scale, since 69.6% of the cohort is Aβ-positive, and are included to bound the worst case: discrimination is preserved, while the 0.5 probability threshold is displaced and accuracy falls accordingly. AUC, area under the receiver operating characteristic curve.

Supplementary Table S4. Cost model unit costs and assumptions.

Cost component	Unit cost	Applied to
Plasma p-Tau217, single analyte	$350	Every patient, except under universal Aβ-PET
Multi-analyte reflex add-on	$250	Only patients routed to Stage 2
Aβ-PET scan	$3,000	Universal strategy: every patient. Staged strategies: indeterminate cases only
Simulated cohort size	10,000	All strategies

Unit costs were drawn from 2024 U.S. Medicare clinical laboratory and physician fee schedules (references 43-45). Where a range was plausible we adopted the higher plasma figure and a conservative Aβ-PET price so that savings are not overstated. Costs are undiscounted, expressed in 2024 U.S. dollars, and exclude clinician time, downstream treatment, and the consequences of misclassification.

Supplementary Table S5. Diagnostic performance of GRAD by stage and cohort.

Cohort and stage	n	AUC (95% CI)	Sensitivity, %	Specificity, %	PPV, %	NPV, %	LR+	LR-	Brier
ADNI, Stage 1	96	0.939 (0.866-0.995)	94.9	91.9	94.9	91.9	11.71	0.06	-
ADNI, Stage 2	49	0.781 (0.630-0.913)	76.9	73.9	76.9	73.9	2.95	0.31	0.191
ADNI, full pipeline	145	0.915 (0.859-0.961)	89.4	85.0	89.4	85.0	5.96	0.12	0.108
A4 + LEARN, Stage 1	1,014	0.904 (0.884-0.923)	82.8	94.1	97.0	70.1	13.99	0.18	-
A4 + LEARN, Stage 2	630	0.793 (0.753-0.832)	66.7	80.0	88.1	51.8	3.33	0.42	0.201
A4 + LEARN, full pipeline	1,644	0.879 (0.861-0.895)	76.7	88.6	93.9	62.3	6.71	0.26	0.145

Stage 1 rows cover only the participants that stage resolves; its sensitivity and specificity use the 0.25 and 0.75 routing thresholds as the decision rule. Stage 2 rows cover the gray zone. Stage 2 and full-pipeline values use a 0.5 probability threshold. Confidence intervals are percentile bootstrap over participants (2,000 resamples). ADNI values are leave-one-out cross-validated; A4 + LEARN values are from the ADNI-fitted model applied without refitting. AUC, area under the receiver operating characteristic curve; LR, likelihood ratio; NPV, negative predictive value; PPV, positive predictive value.

Supplementary Table S6. Operating-point sweep in the ADNI development cohort.

Operating point	Probability threshold	Sensitivity, %	Specificity, %	PPV, %	NPV, %	LR+	LR-	Accuracy, %
Rule-out (90% sensitivity target)	0.451	91.8	81.7	87.6	87.5	5.01	0.10	87.6
Rule-in (90% specificity target)	0.667	81.2	90.0	92.0	77.1	8.12	0.21	84.8

Both operating points are derived in the ADNI development cohort (n = 145) from leave-one-out cross-validated predictions of the full pipeline, and are reported to show the range over which the model can be tuned. They have not been validated prospectively and should not be adopted as fixed thresholds in other populations without recalibration. LR, likelihood ratio; NPV, negative predictive value; PPV, positive predictive value.

Supplementary Table S7. Stage 2 associations and model coefficients.

Feature	Univariable OR per SD (95% CI)	P	Multivariable OR per SD (95% CI)	Penalised coefficient
APOE ε4 carrier	3.14 (1.53-6.44)	.002	2.62 (0.92-7.51)	+0.442
p-Tau217	2.34 (1.20-4.56)	.013	8.16 (1.10-60.54)	+0.344
Aβ42/40	0.32 (0.13-0.77)	.011	0.16 (0.02-1.06)	-0.329
tau-Aβ42/40 ratio	1.80 (0.94-3.47)	.078	0.12 (0.01-1.16)	+0.072
Age	0.58 (0.31-1.10)	.097	0.40 (0.12-1.35)	-0.205
GFAP	0.80 (0.43-1.48)	.482	0.41 (0.05-3.07)	-0.104
GFAP × p-Tau217	0.94 (0.53-1.67)	.829	2.50 (0.30-20.59)	-0.042

All estimates are from the ADNI gray zone (n = 49). Univariable odds ratios fit each feature alone and are the values quoted in Section 3.4. Multivariable odds ratios fit all seven features together without penalisation and are reported so that the two can be compared directly; their intervals are wide because the features are correlated and the sample is small. The final column gives the L2-penalised coefficients of the deployed Stage 2 model on standardised features, whose magnitudes are plotted in Figure 2E. The negative coefficient for Aβ42/40 reflects that a lower ratio indicates greater amyloid burden. The univariable estimates and the penalised coefficients agree in ranking APOE ε4, p-Tau217 and Aβ42/40 highest. The unpenalised multivariable estimates are unstable, with p-Tau217 and the tau-Aβ42/40 ratio taking large coefficients of opposing sign because the two are collinear by construction; this instability is why the deployed model is L2-penalised. OR, odds ratio.

Supplementary Table S8. Feature ablation in the A4 + LEARN gray zone.

Model	Gray zone AUC	Δ AUC vs full model
Full seven-feature model	0.793	-
Without APOE ε4 carrier	0.758	-0.034
Without Aβ42/40	0.763	-0.030
Without p-Tau217	0.773	-0.020
Without tau-Aβ42/40 ratio	0.792	-0.001
Without GFAP × p-Tau217	0.793	+0.000
Without Age	0.797	+0.004
Without GFAP	0.800	+0.008

Each row refits Stage 2 on the ADNI gray zone with the stated feature set and scores the A4 + LEARN gray zone (n = 630) without refitting. A negative Δ AUC indicates that removing the feature degraded external discrimination.

Supplementary Table S9. Predictive values across assumed Aβ prevalence.

Assumed Aβ prevalence, %	PPV, %	NPV, %
20	62.7	93.8
30	74.2	89.9
50	87.0	79.2
70	94.0	61.9

Positive and negative predictive value depend on how common Aβ-positivity is in the population being tested, whereas sensitivity and specificity do not. Values are derived from the full-pipeline operating point in A4 + LEARN (76.7% sensitivity, 88.6% specificity) applied at each assumed prevalence by Bayes theorem. The 30% and 50% rows approximate a primary care memory complaint population and a specialist memory clinic respectively; the observed prevalence was 69.6% in A4 + LEARN and 58.6% in ADNI. NPV, negative predictive value; PPV, positive predictive value.

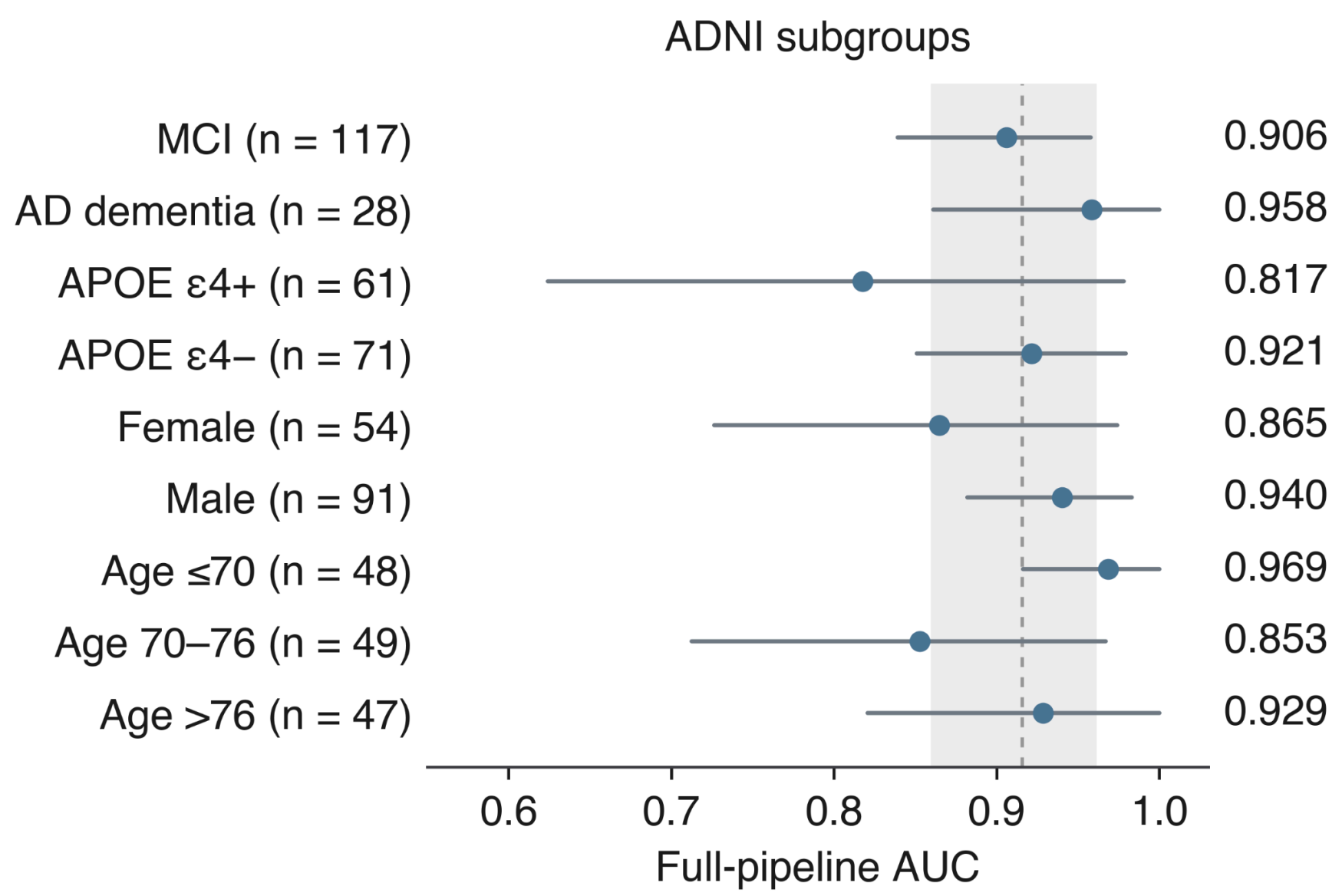

Supplementary Figure S1. Full-pipeline discrimination by subgroup, ADNI development cohort. Area under the receiver operating characteristic curve for the complete GRAD pipeline within clinical subgroups of the ADNI development cohort (n = 145), with 95% percentile bootstrap confidence intervals. The dashed line and shaded band show the whole-cohort estimate and its interval. Intervals are wide for the smaller strata, notably AD dementia (n = 28) and APOE ε4 carriers (n = 61), and should be read as imprecision rather than instability. The corresponding analysis in the A4 + LEARN validation cohort is Figure 3A; cognitive status cannot be stratified there because all participants are cognitively unimpaired.
